# ASSOCIATION OF SOCIAL SUPPORT AND LONELINESS WITH FRAILTY AND FUNCTIONAL STATUS

**DOI:** 10.1093/geroni/igac059.1020

**Published:** 2022-12-20

**Authors:** Kay Thwe Kyaw, Thomas Flagiello, Alec Levine, Joel Salinas

**Affiliations:** NYU Grossman School of Medicine, Hartsdale, New York, United States; NYU Grossman School of Medicine, New York, New York, United States; NYU Grossman School of Medicine, New York, New York, United States; NYU Grossman School of Medicine, New York, New York, United States

## Abstract

Observational studies suggest psychosocial factors such as social support and loneliness are associated with vulnerability for frailty in older adults, but less is known about the generalizability of these putative psychosocial mechanisms in underrepresented groups. Thus, we evaluated whether better telecommunication social support and lower levels of loneliness were associated with decreased frailty and increased functional ability using a unique longitudinal cohort of rural South African older adults. We conducted generalized estimating equation and robust regression analyses using cross-sectional data from 347 participants in the HAALSI (Health and Aging in Africa: A Longitudinal Study of an INDEPTH Community in South Africa) Dementia Cohort. Social support via telecommunication and self-reported loneliness were measured using standard assessments and modeled as exposure variables. Outcomes were frailty (measured using Fried’s frailty phenotype) and functional status (measured by instrumental activities of daily living, IADLs). Lower level of telecommunication social support was associated with higher impairment in ability to perform IADLs. This association persisted after additional adjustments for depression (beta -0.007, 95 % CI -0.016, 0.001) and vascular risk factors (beta -0.012, 95 % CI -0.020, 0.003). No associations were observed in relating telecommunication social support and loneliness with frailty. Among rural South African Black older adults, lower telecommunication-based social support was associated with greater risk of impairment in functional ability. Although further validation is required for possible reverse causality, our findings suggest that future intervention studies focused on promoting telecommunication-based social support to preserve independent functioning may be merited.

